# SIGNIFICANT PREDICTORS OF INTEREST IN GERIATRICS AMONG LOW, MIDDLE, AND HIGH INCOME COUNTRIES

**DOI:** 10.1093/geroni/igz038.539

**Published:** 2019-11-08

**Authors:** Grace Karikari, David K Lohrmann, Lesa L Huber

**Affiliations:** Indiana University School of Public Health, Bloomington, Indiana, United States

## Abstract

Even though the demands for physicians with geriatric related specialties are higher than the available experts around the world, some countries and economies seem to be more impacted by the workforce shortage than others. This systematic review consolidated scientific evidence reporting the significant predictors of interest in pursuing a geriatric career among medical students’ in different countries. A 20-year (1998 – 2018) systematic literature review of high-quality primary research articles was conducted using PubMed, ERIC (EBSCO), Embase and Cochrane Library. Eleven articles (n =11) met the eligibility and quality assessment criteria. For analysis, studies were categorized into (i) low- and middle-income countries and (ii) high income countries, based on the WHO and the World Bank’s income grouping for the 2019 fiscal year. Medical students involved in this review were n = 1,683 representing students from different fields of medicine and year groups. The two most significant predictors of interest in geriatrics in the high-income countries were (1) positive attitudes towards the elderly and (ii) participation in a geriatric related intervention. Lack of published peer-reviewed articles from the low and middle-income countries limited the researchers’ ability to evaluate the similarities and differences between the two income groups. There is an overall need to stimulate interest in geriatric specialization among medical students. Leaders of the LMICs should invest in geriatric education and research in order to promote interest in the field, increase the geriatric workforce, and ultimately, improve the quality of life of the elderly in their respective countries

